# A Comprehensive Review of Climate Change and Plant Diseases in Brazil

**DOI:** 10.3390/plants13172447

**Published:** 2024-09-01

**Authors:** Francislene Angelotti, Emília Hamada, Wagner Bettiol

**Affiliations:** 1 Embrapa Semi-Arid, Brazilian Agricultural Research Corporation, Petrolina 56302-970, Brazil; 2 Embrapa Environment, Brazilian Agricultural Research Corporation, Jaguariúna 13918-110, Brazil; emilia.hamada@embrapa.br (E.H.); wagner.bettiol@embrapa.br (W.B.)

**Keywords:** pathosystems, disease risk, global warming, food security, adaptation, crop protection

## Abstract

Analyzing the impacts of climate change on phytosanitary problems in Brazil is crucial due to the country’s special role in global food security as one of the largest producers of essential commodities. This review focuses on the effects of climate change on plant diseases and discusses its main challenges in light of Brazil’s diverse agricultural landscape. To assess the risk of diseases caused by fungi, bacteria, viruses, oomycetes, nematodes, and spiroplasms, we surveyed 304 pathosystems across 32 crops of economic importance from 2005 to 2022. Results show that diseases caused by fungi account for 79% of the pathosystems evaluated. Predicting the occurrence of diseases in a changing climate is a complex challenge, and the continuity of this work is strategic for Brazil’s agricultural defense. The future risk scenarios analyzed here aim to help guide disease mitigation for cropping systems. Despite substantial progress and ongoing efforts, further research will be needed to effectively prevent economic and environmental damage.

## 1. Introduction

Worldwide efforts have been made in the last decade to reduce greenhouse gas emissions and, consequently, to control both the increase in average air temperature and other extreme events [1]. According to the Intergovernmental Panel on Climate Change (IPCC) [2], global warming is projected to continue increasing in nearly all considered scenarios and modelled pathways in the near term (2021–2040); the best estimates for when the level of global warming of 1.5 °C (relative to 1850–1900) is reached lie in the near term. In the long term (2081–2100), the assessed best estimates and very likely ranges (90–100% probability) of warming vary from 1.4 °C in the very low greenhouse gases (GHG) emissions scenario (SSP1-1.9) to 2.7 °C in the intermediate GHG emissions scenario (SSP2-4.5) and 4.4 °C in the very high GHG emissions scenario (SSP5-8.5). One of the imminent impacts of these climate changes is related to plant health due to the increased risk of introducing new phytosanitary problems [3].

The occurrence of phytosanitary problems caused by diseases, pests, and weeds stands out as one of the factors that will have a potential impact on food security [4,5,6,7,8,9,10,11,12,13,14,15,16,17]. Thus, improving the scientific basis for phytosanitary policy planning, intensifying the national and international dialogue, and raising awareness of the growing impact of climate change and its risks related to plant health are topics of global interest in sustainable development [18,19,20,21,22,23,24,25,26,27,28,29,30,31,32,33,34].

A milestone in this effort was FAO’s International Year of Plant Health (IYPH) in 2020, which was considered a lifetime opportunity to raise global awareness of how protecting plant health can alleviate hunger, reduce poverty, protect the environment, and boost economic development.

The publication entitled “The summary for policymakers of the report on the impact of climate change on plant pests: a global challenge to prevent and mitigate plant pest risks in agriculture, forestry and ecosystems” [3] comprised taking into account the IYPH, focusing on the improvement of international scientific bases for plant protection, and strengthening both phytosanitary policy planning and the legal structures of various organizations.

Faced with this global concern and given the importance of this theme and the need for an internationalization of the content for a wide discussion by international bodies, we decided to prepare a review of all the studies on climate change and plant diseases in Brazil. In addition, the review also aims to identify the main gaps and the main research challenges for plant protection, considering climate change scenarios in the country, in order to strengthen management strategies to reduce losses caused by phytosanitary problems. This document presents studies carried out in Brazil not only through simulation and analysis, but also through experimentations both in controlled and in field conditions. As most studies have been published in Portuguese, they are not readily available internationally. The criterion adopted to compile this review was to encompass all publications addressing climate change and plant diseases in Brazil. We conducted searches across databases including Web of Science, Google Scholar, and SciELO (Scientific Electronic Library Online). Furthermore, we thoroughly reviewed all Brazilian agricultural scientific journals to ensure comprehensive coverage of all relevant studies that may have been absent in the databases previously mentioned.

## 2. Plant Disease

According to Gäumann [35], plant disease is a dynamic process in which a host and a pathogen, closely related to the environment, are mutually influenced, resulting in morphological and physiological changes. The classic disease triangle [36], formed by susceptible host, virulent pathogen, and favorable environment, defines the conditions for the development of diseases caused by biotic agents such as fungi, bacteria, virus, nematodes, oomycetes, etc. [37,38]. These three components are indispensable in the occurrence of a disease.

The environmental factors (temperature, humidity, wind, leaf wetness, UV radiation) and the environmental manipulations (fertilizers, residues incorporation, water supply, greenhouse and hydroponic systems, etc.) affect the incidence and severity of the disease [21,22,39,40,41,42,43,44,45,46,47,48,49,50,51,52,53,54,55,56,57,58]. Since environments and diseases are closely related, climate change is likely to alter the geographical and temporal distribution of plant diseases [59,60,61,62,63,64,65,66,67,68,69,70,71,72]. The impacts of climate change on plant disease can be positive, negative, or neutral, since these changes can decrease, increase, or have no impact on diseases, depending on the region or period [73,74]. These changes also have consequences on the control of plant diseases, through the use of resistant varieties, chemical, biological and cultural control, and mainly through the management of plant diseases. Therefore, for the management of plant disease, knowledge of the potential impacts, considering future scenarios of climate change, is essential to avoid serious losses. In addition to allowing for the adaptation of existing chemical, biological and cultural control methods, this knowledge will also allow for the development of new resistant cultivars, thereby preventing food supply problems caused as a result of climate change [38,59,74,75].

The effects of climate change, such as increases in temperature and atmospheric CO_2_ concentrations as well as changes in rainfall patterns, are studied and discussed both for soilborne plant diseases and those occurring in the aerial part of the plant [44,76,77,78,79,80,81,82,83,84,85]. Climate change, according to Wakelin et al. [80], may affect the natural lifecycle of plant pathogens, influence host predisposition to infection or disease expression, shift the place in which pathogens occur, and alter the rate of genetic change in pathogen populations.

The effects of climate change on plant diseases will not be similar in all regions and crops but will differ for each pathosystem in specific regions of the world. Climate change will interfere with the geographical and temporal distributions of plant disease; thus, existing control methods should be adapted [59]. Strategies for disease management are adapted depending on climate conditions, which requires continuous assessment regarding efficiency [38].

In the new scenarios, the use of fungicide will certainly change [86]. The most important changes may occur due to pressure from society for a reduction in the use of chemical pesticides and an increase in the use of non-chemical methods to control plant diseases [59]. The dynamics of fungicides in plants (penetration, translocation, and degradation) can undergo changes due to changes in temperature and precipitation as well as both morphological and physiological changes. Discussions over the effects of climate change on fungicide use were highlighted by several authors [38,59,74,87,88,89,90,91,92,93]. Important consequences of climate change in the pathogen–host relationship are related to the genetic resistance of plants to pathogens [94,95,96]. Changes in morphology and physiology can alter the resistance mechanisms of cultivars [97,98,99,100].

Biocontrol agents are microorganisms that co-occur on all plant parts [101,102]. Climate change is likely to affect both the diversity of such bioagents and the ways in which they interact with the host, pathogen, and other microorganisms on the plant [87]. Climatic changes can alter the composition and the dynamics of the microbial community of the soil and the aerial environment sufficiently to influence plant health [87]. Consequently, considering natural, conservation or augmentative biological control, changes in the microbial community of the phyllosphere, rhizosphere, spermosphere, and carposphere can influence the occurrence of plant disease [103,104].

Natural biological control is an ecosystem service with which the disease is naturally controlled without any human intervention [105,106,107]. Consequently, the balance of the microbial populations will be altered along with a possible alteration in the natural biocontrol. Conservation biological control consists of human actions that protect or stimulate the performance of naturally occurring natural enemies [107]. The induction of soil suppressiveness to soilborne plant pathogens is an important example of conservation biological control, and has been continuously expanding, especially as a result of greater knowledge related to the ability of plants to recruit certain groups of organisms [108,109,110,111,112,113,114].

Augmentative biological control concerns the periodic release of natural enemies [115]. The efficacy of biocontrol agents released periodically can vary depending on environmental conditions [103]. It is possible that certain species of agents used in this type of control do not perform efficiently in conditions characterized by the predicted increase in temperature [103].

*Coniothyrium minitans* and *Clonostachys rosea*, used to control *Sclerotinia* and *Botrytis*, respectively, are sensitive to increased temperatures since they are efficient within a narrow temperature range. However, other organisms, such as Bacillus spp. are not significantly impacted by climate change since they are effective over a wide temperature range [116,117,118].

The effects of climate change on plant diseases have been studied for the past two decades [119,120,121,122,123,124,125,126,127,128,129,130,131,132,133,134,135,136]. However, limited information is available regarding the effects of these changes on biocontrol agents of plant disease. Thus, considering climate change is crucial during the process of isolation and in the selection of antagonists. According to Research and Markets [137], the global market for biopesticides is projected to reach US$11.3 billion by 2027 and is estimated at US$5.5 billion in 2022. During this forecast period, a compound annual growth rate (CAGR) was estimated at 15.5%. Considering that Brazil treats the largest area under biological control in the world [138], it is essential to dedicate more efforts to assess the effects of climate change on biocontrol agents.

## 3. Impacts of Climate Change on Food Security

**Figure 1:**
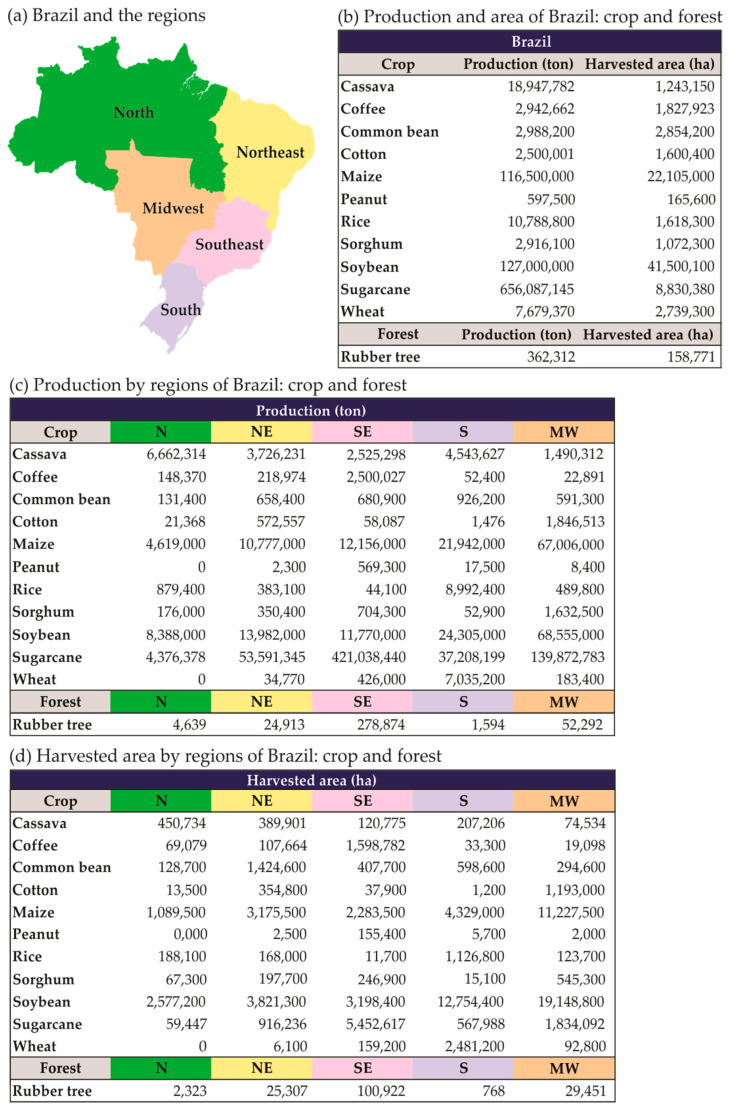
Production of crops and forest (ton) and harvested area (ha) of Brazil and distributed by regions (North—N; Northeast—NE; Southeast—SE; South—S; and Midwest—MW) of Brazil. Productions estimated for 2021/2022 harvest season, except for rubber tree in 2019; peanut in 2020/2021 harvest season; and cassava, coffee, and wheat in 2021. {Data from AGRIANUAL [139]}.

**Figure 2:**
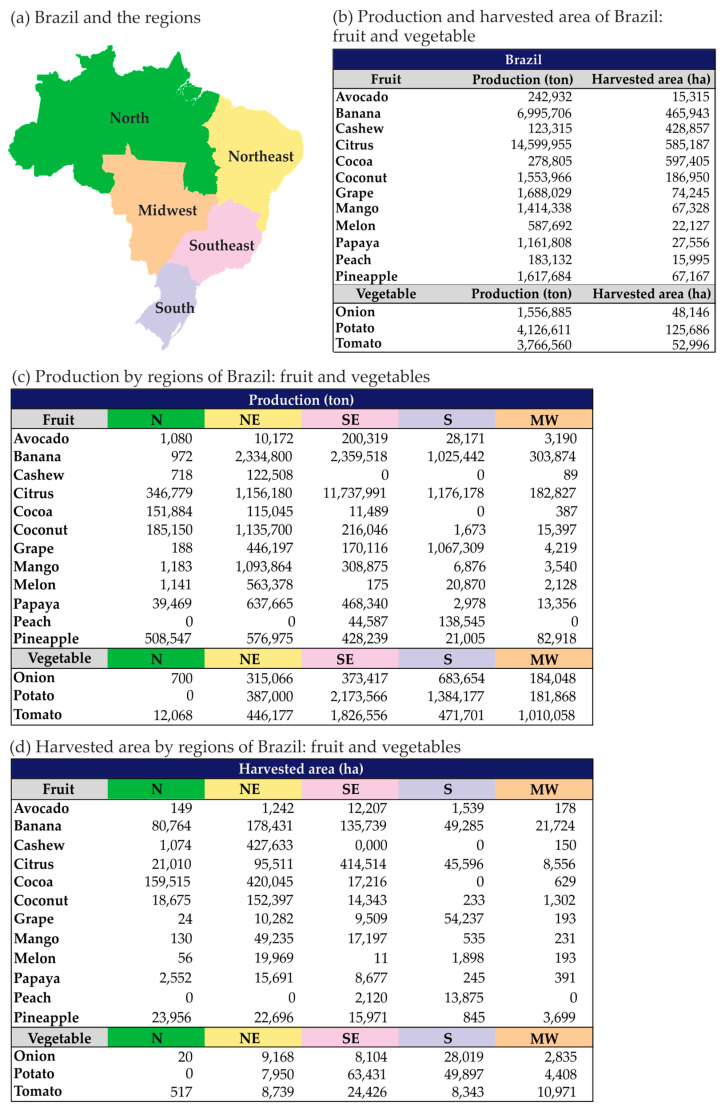
Production of fruits and vegetables (ton) and harvested area (ha) of Brazil and distributed by regions (North—N; Northeast—NE; Southeast—SE; South—S; and Midwest—MW) of Brazil. Productions in 2019, except for banana, cashew, citrus, cocoa, grape, potato, and tomato in 2021. {Data from AGRIANUAL [139]}.

The importance of Brazilian agricultural production for global food security is shown in Figure 1 and Figure 2. Thus, it is imperative to gather extensive information on the impacts of climate change on the occurrence of diseases in major Brazilian commodities, including soybean, coffee, sugarcane, corn, cotton, and orange, as well as in fruits and family farming products such as banana, cassava, common bean, vegetables, and others.

Brazil’s soybean production for 2021/2022 harvest season was 127 million tons (Figure 1). As the leading producer of soybean globally, Brazil’s production accounts for approximately 36% of the total output in 2021 [140,141,142]. Considering the product’s role in ensuring global food security, Brazil’s soybean production is of great importance.

The worldwide production of sugarcane in 2021 was 1.859 billion tons, with Brazil contributing approximately 38% of the world’s output, making it the world’s largest producer [140]. In the 2021/2022 harvest season, the production volume was 656 million tons (Figure 1). The harvest is used both for sugar and ethanol production. Notably, ethanol plays a crucial role in the replacement of fossil fuels in the country [143,144,145,146,147].

Furthermore, Brazil is the world’s largest producer of coffee, with an output volume of nearly 2.94 million tons in the 2021/2022 harvest season (Figure 1). It is worth noting that the production of these crops is highly vulnerable to climatic conditions, which in recent years have shown strong variations such as droughts and frosts [148,149,150,151,152,153].

Thus, reductions in production and productivity caused in a climate change scenario will impact food security globally, particularly fruit and vegetable crops, not only due to physiological problems, but also as a result of diseases and pests [4,5,154]. Soybean, coffee, and sugarcane plantation crops are susceptible to a wide range of diseases and pests with the potential to reduce crop productivity [155,156,157,158,159,160,161,162,163,164,165,166,167,168,169,170,171,172]. Given the environmental sensitivity of plant pathogens and pests, which pose important threats to crop output, it is imperative to know the potential impact of climate change on the severity and incidence of diseases and pests in Brazil.

Brazil is not only renowned for its production of coffee, sugarcane, and soybeans, but it also plays a significant role in the production of corn and cotton (Figure 1). Additionally, the country is a major producer of cassava, common bean, potato, rice, and fruits (Figure 1 and Figure 2), which are crucial components of the Brazilian diet. Thus, it is imperative to conduct regionalized studies based on the dynamics of phytosanitary problems in different producing areas and on future climate change scenarios.

## 4. The Impacts of Climate Change on Plant Disease

In tropical regions, including South America, the projections of climate seasonality under climate change are still uncertain, particularly concerning precipitation and temperature [173]. Based on the Coupled Model Intercomparison Project (CMIP), a comparison between future temperature projections and observations of CMIP3 (which was used as a base for the 3rd and 4th IPCC Assessment Reports), CMIP5 and CMIP6 (base for the latest release) shows that the global warming projected by these CMIPs and future climate scenarios analyzed indicates a slightly lower global warming level when compared to the observed one [174]. Furthermore, the observed warming is closer to the upper level of the projected future climate scenarios, revealing that the CMIPs with higher GHG emissions appear to be the most realistic.

**Figure 3:**
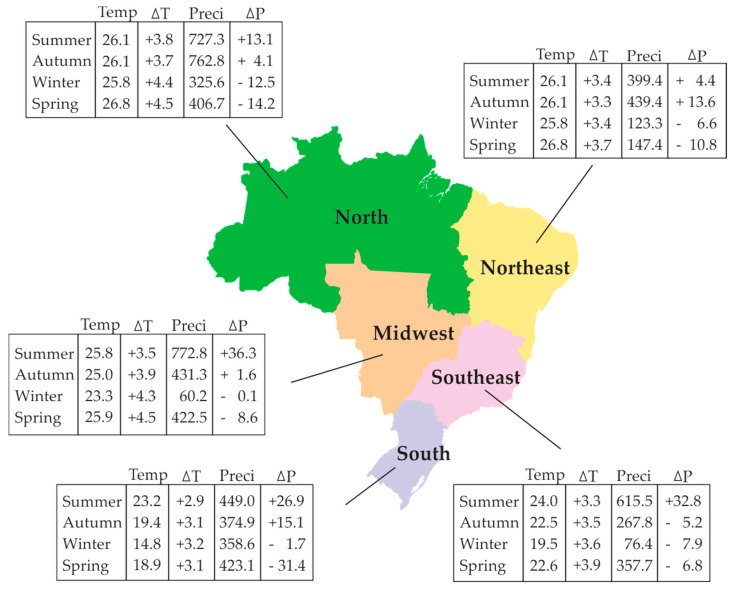
Average air temperature—Temp (°C) and average daily precipitation Preci (mm) by season in Brazil from 1961 to 1990; and anomaly of temperature (ΔT) and precipitation (ΔP) compared to the period 2071 to 2100, scenario A2. Summer (December, January, February), Autumn (March, April, May), Winter (June, July, August), and Spring (September, October, November). {Data from Hamada et al. [175]}.

The climatic diversity in Brazil can be observed in Figure 3, which illustrates the seasonal variations in temperature and precipitation across the five regions of the country. These variations are significantly shaped by Brazil’s continental nature [175,176]. Figure 3 displays the seasonal mean of observed air temperatures and precipitation from 1961 to 1990, as well as projected temperature (ΔT) and precipitation (ΔP) anomalies from 2071 to 2100 for scenario A2 of the 4th IPCC Report relative to the baseline period of 1961 to 1990. Scenario A2 is considered the most pessimistic scenario. This scenario describes a very heterogeneous world with a continuously increasing global population, per capita economic growth, and technological change that is more fragmented and slower compared to other scenarios, among other features [177].

Due to the fact that the studies covered here considered projections from the 3rd and 4th Reports, we present the data based solely on the 4th Report. It is worth noting that the data from the 4th Report differ slightly from more recent IPCC Reports [174].

The North and Northeast regions have an average temperature of around 26 °C, with a forecasted increase in temperature between 3.3 and 4.5 °C for the period between 2071 and 2100. The South and Southeast regions are characterized by winters with average temperatures of 14.8 and 19.5 °C, respectively. In these regions, the warmest months have average temperatures ranging between 23.2 and 24 °C, with a forecasted increase of up to 3.5 °C for the summer. In the Midwest region, the average temperature varies between 23.3 and 25.9 °C, with a forecasted increase of up to 3.2 °C (Figure 3).

In order to forecast the potential impacts of climate change on the main diseases in different regions of Brazil, Brazilian experts used future climate data based on the 3rd and 4th IPCC reports, compiled from Ghini and Hamada [178], and Ghini et al. [75], respectively, as shown in Table 1.

This summarized information (Table 1) shows that, for the diseases that affect aerial parts, climate change will increase the severity of anthracnose in maize, sorghum, cashew, mango, melon, onion, papaya, stone fruit, and strawberry (during rainfall). On the other hand, such severity will decrease for cassava (in the North, Northeast and Midwest regions), pepper, rubber tree, and strawberry (fruit rot), while it will remain the same for cassava (in the South and Southeast regions) pepper and grapes. The severity of powdery mildews will increase for cassava (in the South), winter cereals, cashew, grape (for some regions), mango, rubber tree, lettuce, tomatoes, pepper, and strawberries; with the severity expected to be reduced for papaya, and remain stable for eucalyptus, melon, and grapes. For downy mildew, severity will increase for maize, melon, and lettuce; reduce for sorghum, brassicas, and onion; and remain stable for grape. For rust, severity will increase for coffee, sorghum, and stone fruit; reduce for maize and soybean; and remain stable for sugarcane, winter cereals, eucalyptus, and grape. For root rot caused by the several pathogens shown in Table 1, severity tends to increase for lettuce, maize, sorghum, stone fruit, and strawberry; reduce for cassava and pineapple; and remain the same for winter cereals. When considering the Fusarium genus, the severity tends to increase for maize, rice, sorghum, banana, pineapple, strawberry, lettuce, onion, and tomato; remain the same for mango, winter cereals, cassava, and maize; and reduce for papaya. Diseases transmitted by vector will be discussed further.

Studies using maps of the geographic and temporal distribution of climate favorability for the occurrence of plant diseases were created to evaluate the risks of climate change (Table 2). These studies were carried out for fifteen pathosystems of eight crops (banana, cacao, coffee, common beans, eucalyptus, grape, papaya, and peanut). The available climatic information on disease occurrence in the literature served as basis for the forecast. The results showed that nearly half of the pathosystems exhibited an increase in favorability under predicted future climate conditions, whereas the remaining demonstrated a reduction. Nevertheless, some diseases indicated stability depending on the region.

Studies on the effects of elevated CO_2_ in the incidence and severity of diseases in five crops (coffee, eucalyptus, melon, rice, and soybean) were conducted under controlled conditions and the results are shown in Table 3. Among these studies, three were carried out under phytotron conditions [225,226,227], six were conducted in open-top chambers [73,228,229,230,231,232,233], and only two studies [76,126] were carried out in a free-air CO_2_-enrichment (FACE) facility in field conditions. It is widely known that FACE facilities provide more realistic conditions with which to understand how CO_2_ influences plant performance, including disease responses; however, the installation and maintenance of these facilities are costly. As shown in Table 3, the severity of coffee leaf rust, caused by *Hemileia vastatrix*, was reduced under elevated CO_2_ levels, as observed in studies conducted in both FACE [76,126] and open-top chamber conditions [231]. Likewise, an open-top chamber study demonstrated a reduction in disease severity for eucalyptus rust (*Puccinia psidii*) [73]. Regarding rice, studies also conducted under open-top chambers demonstrated that crops affected by rice blast caused by *Magnaporthe oryzae* had an increase in disease severity ([230], while crops affected by brown spot, caused by *Bipolaris oryzae* experienced a reduction [232,233]. All the abovementioned crops showed an increase in plant growth under elevated levels of CO_2_.

Brazil has a diverse agricultural production that spans across a vast territory with varying climates (Figure 3), encompassing both temperate and tropical plants. In addition, there is a wide range of plant pathogens (Table 1, Table 2 and Table 3). The present study considered 304 pathosystems, covering 32 crops of economic importance for the country. The causal agents studied were fungi, bacteria, viruses, oomycetes, nematodes and spiroplasm (Table 1, Table 2 and Table 3), with fungi being the focus of approximately 79% of the studies conducted to date due to their significance.

It is concluded that, while some diseases may lose significance or even maintain a steady state, almost 46% of the diseases considered will gain importance in Brazil’s future climate scenario (Table 1, Table 2 and Table 3).

**Figure 4:**
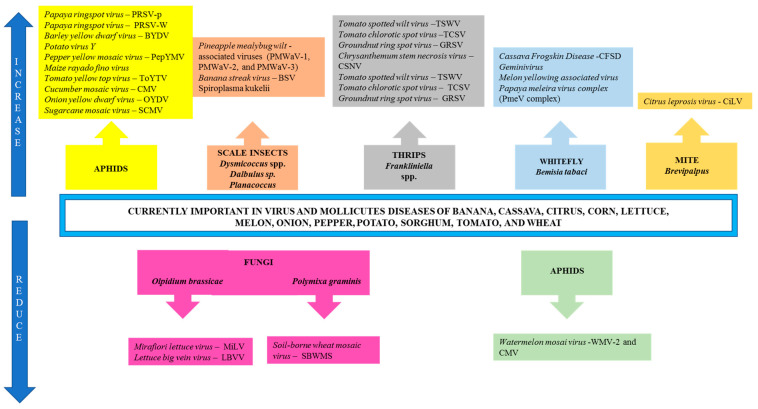
Potential impact of global warming on virus and mollicutes diseases in Brazil transmitted by vectors {data from Ghini and Hamada [178] *; and Ghini et al. [209]}. (*) The English version of this reference was published in Ghini and Hamada [210].

Diseases in potato, tomato, pepper, melon, corn, banana, and citrus, caused by viruses and mollicutes and transmitted by vectors, were described in Ghini and Hamada [210], while those in lettuce, onion, papaya, cassava, and sorghum, caused by viruses transmitted by insect vectors, were discussed in Ghini et al. [38]. With global warming, vectors will have shorter lifecycles, greater longevity, and higher activity, which will lead to an increase in their population and importance in all regions of Brazil, making such diseases more prevalent (as shown in Figure 4).

## 5. Diseases Transmitted by Vectors

Global warming will play an important role in the increase in the population of vectors that carry viruses and mollicutes, which are responsible for diseases in potato, tomato, pepper, melon, winter cereal, maize, banana, and citrus [210], as well as onion, papaya, cassava, sorghum, and lettuce [209]. These vectors will have shorter lifecycles and activity.

*Potato leafroll virus* (PLRV) and *Potato virus Y* (PVY), the main potato viruses, are both transmitted by aphids. A higher temperature should encourage epidemics of these two viruses due to an increase in the movement of vectors and a decrease in the reproduction cycle [206].

*Tomato viral wilt*, caused by *Tospovirus* genus [*Tomato spotted wild virus* (TSWV), *Tomato chlorotic spot virus* (TCSV), *Groundnut ring spot virus* (GRSV), and *Chrysanthemum stem necrosis virus* (CSNV)], transmitted by thrips (*Frankliniella fusca*, *F*. *intonsa*, *F*. *occidentalis*, *F*. *schultrzei*, *F*. tenuicornis, *Scirtothrips dorsalis*, *Thrips palmi*, *T*. *setosus*, and *T*. *tabaci*); *tomato golden mosaic*, caused by more than 14 different species of *Geminivirus* and transmitted by whitefly (*Bemisia tabaci* biotipo B), and *Potato virus Y*, *Pepper yellow mosaic virus* (PepYMV), *Tomato yellow top virus* (ToYTV), and *Cucumber mosaic virus* (CMV), transmitted by aphids, will become more important between March and September in all regions because the increase in temperature will bring an increase in the population of thrips, whitefly, and aphids [208] (Figure 4).

In pepper, viral wilt caused by the *Tospovirus* genus (TSWV, TCSV, GRSV, and CSNV) transmitted by the same species of thrips, as well as PVY, PepYMV, and CMV, transmitted by aphids, shall become more important between March and September in all regions [207].

In melon, *Melon yellowing associated virus* will remain an important virus, transmitted through grafting and whitefly (*Bemisia tabaci* biótipo B). However, the importance of *Papaya ringspot virus* (PRSV-W), *Watermelon mosaic virus* (WMV-2 and CMV), despite being transmitted by aphids, may decrease due to an expected increase in precipitation [198] (Figure 4).

Winter cereals are expected to experience an increase in the importance of *Barley yellow dwarf virus*—BYDV, which is transmitted by aphids. On the other hand, the importance of *Soil-borne wheat mosaic virus* (SBWMS), transmitted by the fungus *Polymixa graminis*, is expected to decrease [186]. For maize, there is an expected increase in the importance of corn stunt spiroplasma, caused by *Spiroplasma kukelii*, and maize bushy stunt phytoplasma, transmitted by scale insects (*Dalbulus maidis*), particularly in the South and Southeast regions between April and July. The same trend can be observed for *Maize rayado fino virus*, *Sugarcane mosaic virus*, and *Maize mosaic virus*, which are transmitted by *D. maidis*, aphids, and *Perigrinus maidis*, respectively [181] (Figure 4).

Banana streak disease, caused by *Banana streak virus* (BSV) and transmitted by scale insects (*Planacocus citri* and *Pseudococcus* sp.) and contaminated propagative material will increase in importance. Such propagative material is related to vegetative material from regions where seedlings are produced through the tissue cultures not used in cultivation [192]. Citrus leprosis, caused by *Citrus leprosis virus*—CiLV, will tend to increase due to an elevated population of the mite *Brevipalpus*, which is associated with a rise in temperature [194] (Figure 4).

Global warming will play an important role in the proliferation of insect vectors that carry the viruses responsible for diseases in onion, papaya, cassava, sorghum, and lettuce. As temperatures increase, the populations of these vectors are expected to experience shorter lifecycles and higher activity levels.

*Onion yellow dwarf virus* (OYDV), transmitted by aphids (*Aphys gossypii*, *Macrosiphum ambrosiae*, and *Myzus persicae*), will increase in incidence due to a rise in the population of its vector caused by climate change [205].

*Papaya ringspot virus* (PRSV-p), which affects papaya and is transmitted by *A*. *gossypii*, and *Papaya meleira virus complex* (PmeV complex), which causes Papaya Sticky Disease and is transmitted by *Bemisia tabaci* biotype b, are also likely to become more important [199].

*Pineapple mealybug wilt-associated viruses* (PMWaV-1, PMWaV-2, and PMWaV-3), which are transmitted by the scale insects, *Dysmicoccus brevipes* and *Dysmicoccus neobrevipes* that are associated with ants, will increase in pineapple in future scenarios [200].

*Sugarcane mosaic virus*, transmitted by aphids in sorghum, and *Cassava Frogskin Disease* (CFSD), transmitted by *Bemisia tuberculate* in cassava, will tend to increase in incidence [179,183].

In lettuce, *Tomato spotted wilt virus* (TSWV), Tomato chlorotic spot virus (TCSV), and *Groundnut ring spot virus* (GRSV), transmitted by thrips (*Frankliniella fusca*, *Frankliniella occidentalis*, and *Thrips tabaci*) will increase in importance. However, the importance of Big Vein in lettuce, which is caused by the *Mirafiori lettuce virus* (MiLV) and *Lettuce big vein virus* (LBVV), and transmitted by the fungi *Olpidium brassicae*, will be reduced (Figure 4). On the other hand, Lettuce mosaic virus (LMV) and *Lettuce mottle virus* (LeMoV), transmitted by aphids, will likely have their importance unaltered (Figure 4).

## 6. Research Gap

In Brazil, 90% of the studies conducted to evaluate the effects of climate change on plant diseases have been carried out with crops of agricultural importance. However, knowledge regarding the impacts on natural systems and planted forests is still very limited.

Risk analyses, generated through geographical and temporal distribution maps, and also through experimentation in field conditions, are used as monitoring tools to validate results and adopt protective measures for the cultivation systems.

In addition to monitoring of the incidence and severity of plant diseases, research will also play an important role in filling some knowledge gaps. It is essential to obtain information on the multitrophic host–pathogen interaction, the breakdown of genetic resistance, the predisposition of plants to climate change, evolutionary adaptation, and mitigation measures for plant protection [168,234,235,236].

Most studies are carried out under controlled conditions with constant temperatures, in which simplified systems are evaluated with individual stresses and, often, a single host plant interacting with a pathogen. However, in natural conditions, plants are exposed to both biotic and abiotic stresses simultaneously [237]. In order to reduce uncertainties and predict the impact of these stresses on plants more accurately, it is important to conduct studies involving a wider set of interactions, as the incidences and severity of plant diseases are complex processes. The behavior of plants in the natural environment shows the differentiated response to multi-pathogen systems, whose interactions include coexistence, cooperation, or competition [238].

Understanding how plants react to increased air temperature and water deficits, as well as to resistance mechanisms, can contribute to reducing the negative impacts of climate change. In some cases, changes in climate conditions require adaptive mutations in plants that may result in ecological costs. One example of this phenomenon can be seen in *Brassica rapa*, in which early flowering in response to water stress caused a reduction in natural defense against *Alternaria brassicae* [239].

Apart from the predisposition to water deficits, increases in temperature also change the evolutionary pressure on plants. Thus, advances in research on genetic resistance aiming at the search for thermostable genes may include epigenetic factors that are still incipient in cultivated plants [240]. In addition to these advances, studies based on species adaptability that occurs either through phenotypic plasticity or genetic adaptation, will be imperative [241,242]. As knowledge is a continuous process, a great challenge lies in the integration of this information into mathematical models and into tools to assess the impact of climate change that will enable the development of strategies to protect plants against the adverse effects of future climate scenarios.

This review shows that the studies carried out in Brazil, until now, have been based on the assessment of impacts on phytosanitary problems. Many of these studies report the need for adaptation measures that have not yet been effectively adopted. For example, Kobori et al. [204] predicted that the importance of downy mildew in lettuce would decrease in summer and increase in winter. However, the same authors observed that the causal agent (*Bremia lactucae*) has undergone adaptations to the rising temperatures of recent years, leading to an increase in the importance of the disease during the summer.

## 7. What to Do after Risk Assessment

Based on the analysis of the impacts of climate change on the occurrence and severity of plant diseases, different responses of pathosystems to important agricultural crops in Brazil have been identified. The responses include the increase in risk, reduction in risk, and maintenance of risk (Table 1, Table 2 and Table 3). Since around 50% of the phytosanitary problems analyzed in this study presented an increased risk due to climatic favorability, the management of plant diseases will continue to play a fundamental role in the crops of economic importance for both Brazil and the world, taking food security into account. Furthermore, scenarios with lower risk have direct implications for phytosanitary management due to a reduction in the need for the chemical application of pesticides and, consequently, reductions in production costs and environmental impacts.

**Figure 5:**
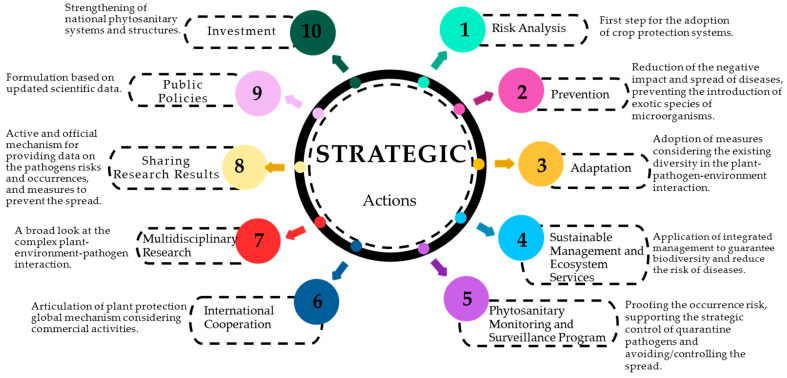
Strategic actions to tackle the impacts of climate change on crop protection systems.

In this way, ten strategic actions are outlined to tackle the impacts of climate change on crop protection systems in Brazil (Figure 5). The first step for the adoption of crop protection systems is carrying out a **Risk Analysis** based on climate change. The risk analyses can be conducted using methods such as geographical and temporal distribution maps and experimentation in controlled and field conditions. This information subsidizes the validation of the results and the adoption of protective measures for cultivation systems.

The next step is **Prevention**, in which the focus lies on a reduction in the negative impact and spread of diseases, and in preventing the introduction of exotic species of microorganisms. According to the IPPC Secretariat [3], prevention is one of the key strategies to avoid economic and environmental damage resulting from the impacts of climate change on the occurrence of phytosanitary problems. Identifying the vulnerabilities of cropping systems before the introduction and establishment of the phytopathogen is an extremely important preventive measure [243]. Human beings are among the most important agents of epidemics through national and international travel and commercialization. Furthermore, the global seed and propagation material market is one of the main contributors to the rapid spread of plant pathogens to new hosts [3,244]. Therefore, responsible practices should be enforced to reduce the spread and dissemination of pathogens.

The adoption of **Adaptation** measures involving the existing diversity in the plant–pathogen–environment interaction is imperative. Long-term measures include, as follows: obtaining tolerant cultivars, new chemical/biochemical molecules, and the selection of bioagents that are effective even in the high temperatures of some regions in Brazil. Short-term adaptation measures include, as follows: integrated technologies that can be adopted through diversified cropping systems; the use of pathogen-free seeds and propagation materials; the adoption of biological control agents, growth promoters, abiotic stress mitigators, mycorrhizal fungi and endophytic microorganisms; the application of physical barriers, solarization, sanitation techniques, efficient irrigation and nutrition; and the support of epidemic alert and forecast systems.

Another strategy includes **Sustainable Management and Ecosystem Services** aiming to ensure biodiversity and contribute to the reduction in disease risks in agricultural and natural systems [50,245,246,247]. Sustainable alternatives include diversified, flexible, and resilient cropping systems [248,249], in which multiple and integrated approaches can reduce vulnerability and contribute to social, economic, and environmental development. Regenerative agriculture, focusing on soil recovery, plays a strategic role both in adopting integrated management and in increasing the biodiversity. An example includes the incorporation of crop residues into the soil to reduce the frequency of *Fusarium* species [250], and the maintenance of earthworm communities with a bioregulatory role in degrading mycotoxins and maintaining soil health for sustainable production [251].

Furthermore, to address the impact of climate change on plant defense, a **Phytosanitary Monitoring and Surveillance Program** is necessary in order to confirm the risk of the occurrence of phytosanitary problems, support the strategic control of quarantine pathogens, and prevent/control the spread of diseases. Establishing monitoring systems to detect the occurrence, and to measure the severity, of diseases is an important maneuver that needs to be performed in regional, national, and international surveillance programs. Joint actions among municipalities, states, and countries play a fundamental role in promoting practices that reduce dissemination. An example of an international global monitoring action is the Borlaug Global Rust Initiative (https://bgri.cornell.edu/, accessed on 10 May 2022), which managed to diagnose the emergence of new strains and issued alerts of possible rust outbreaks [243].

The strategies also include **International Cooperation** through the articulation of a global mechanism of plant protection considering commercial activities. The implementation of regulatory frameworks considering activities involving agricultural products plays a crucial role in the adoption of responsible plant protection practices aiming at the reduction in the spread of microorganisms [3]. These practices also contribute to addressing the global challenges of food security, environmental protection, and economic development. Strategic studies among neighboring countries, such as Brazil and Argentina, showed favorable climatic conditions for the development of sugarcane orange rust (*Puccinia kuehnii*) in the main producing departments in Argentina, where the pathogen has not yet been identified, reinforcing the need to strengthen plant protection actions [252].

Complex host-plant dynamics require **Multidisciplinary Research** involving scientific cooperation among different areas of knowledge for a broad approach to the complex plant-environment-pathogen interaction. Such cooperation is vital to enable fast integration of information, avoiding losses caused by diseases in the context of climate change.

**Sharing Research Results** means exchanging information through an active and official mechanism to provide data on risks, occurrences, and measures to prevent the spread of pathogens. It is necessary to articulate a global mechanism for the protection of plants that considers not only commercial activities but also makes existing knowledge available to avoid loss and damage and consequently reduce food safety risks in this new scenario of climate change. Technological tools facilitate the rapid dissemination of research findings and data on plant health, aiding in global collaboration and knowledge exchange. Unmanned aerial vehicles and the Internet-of-Things are examples of technology applied to phytosanitary monitoring aiming at the detection of phytopathogens, reducing the risk of disease dissemination, and preventing the introduction of exotic species [253,254]. Additionally, recent studies indicate the use of nanomaterials as biosensors for the early diagnosis of plant diseases [255,256], and extending the use of nanomaterials in the control of phytopathogens and as elicitors of the immune systems of plants [256,257]. However, the knowledge of new technologies, especially those at the molecular level, is not universally disseminated.

The formulation of **Public Policies** based on current scientific data is essential for the adoption of technologies aimed at protecting plants. These policies aim to ensure the sustainability of production systems by encouraging the rational handling of pesticides and implementing measures to reduce the spread of pathogens [3]. In Brazil, the Defense Plan plays a crucial role in agricultural defense aiming at the sustainable development of agribusiness [258]. Therefore, aligning scientific developments with the impact of climate change on the occurrence of phytosanitary problems is imperative for advances in sanitary actions.

Finally, it is paramount to highlight the role of **Investment**. By strengthening national phytosanitary systems and structures, we will provide a sturdy foundation for the establishment of a global research support mechanism. Through strategic investment, we will promote scientific innovation tailored to confront the challenges posed by climate change. By doing so, we not only protect the integrity of agricultural systems but also pave the way for a more sustainable future.

## 8. Conclusions

Various research groups from different institutions in Brazil have conducted studies on the impact of climate change on plant diseases. This has enabled the assessment of phytosanitary risk throughout a broad range of pathosystems. The results of this study indicate that climate change will increase the importance of diseases caused by plant pathogens, as can be observed by the rise in 46% of pathosystems considering the timescale between 2001 and 2100. In particular, viruses and mollicutes transmitted by insects and mites to vegetables, fruits, and cereals will be the most affected by the climate change.

Predicting the occurrence of diseases in the face of climate change scenarios is a complex challenge for scientific research and the continuity of this work is strategic for national agricultural defense. This requires continued simulation and field studies that incorporate the adoption of new short- and long-term adaptation strategies and the adaptation of pathogens and crops to climate change. Therefore, advances in this line of research will need to include the monitoring of the occurrence of diseases and the implementation of adaptation measures. As of now, such measures are still incipient or almost non-existent in Brazil.

While the continental nature of Brazil provides advantages for agricultural diversity, greater attention needs to be given to studies on the geographic and temporal distribution of pathogens, particularly regarding their dispersion from tropical regions, such as the Northeast, to temperate regions in the South. Despite considerable effort having already been spent on this topic, some crop diseases, such as those in cotton and avocado crops, have yet to be analyzed. Risk scenarios are crucial in identifying the vulnerability of cropping systems to diseases in climate change scenarios and further scientific advancements are necessary to effectively prevent economic and environmental damage.

## Figures and Tables

**Table 1:** Effects of climate change on future importance of diseases of different pathosystems and in different regions considering current optimal conditions for disease development in Brazil.

Host—Reference	Pathogen (Disease)	Appropriate Environmental Conditions of Temperature, Relative Humidity (RH) and Precipitation for the Occurrence of Diseases	Effects of Climate Change on Future Importance of the Disease in Different Regions
Crops and plantation crops			
Cassava—[179]	*Cercosporidium henningsii* and *Cercospora vicosae* (Brown leaf spot, Diffuse leaf spot)	Rainy season	Remain similar
	*Colletotrichum gloeosporiodes* f. sp. *manihotis* (Anthracnose)	18–28 °C and high RH	Reduce in North, Northeast and Midwest, and will be remain similar in South and Southeast regions
	*Oidium manihotis* (Powdery mildew)	15–35 °C and RH between 85–95%	Increase in South
	*Phaeoramularia manihotis* (White leaf spot)	Mild weather	Remain similar
	*Phytophthora drechsleri* and *Fusarium solani* (Root rot)	Prolonged periods of rain and poorly drained soils	Reduction for *Phytophthora*, except in the Southern region. Remain similar importance for Fusarium
	*Sphaceloma manihoticola* (Superalongation)	20–28 °C and high precipitation	Reduce
	*Uromyces manihotis* (Rust)	18–23 °C and high RH	Reduce in North, Northeast, and Midwest
	*Xanthomonas axonopodis* pv. *manihotis* (Cassava bacterial blight)	20–30 °C and RH > 90%	Increase in Midwest, South and Southeast
	*Cassava Common Mosaic Virus* (CsCMV)	Mild weather	Reduce
	*Cassava Vein Mosaic Virus* (CsVMV)	High temperature	Increase
Coffee—[180]	*Cercospora coffeicola* (Brown eye spot)	18–24 °C and precipitation greater than 3 mm/day	Reduce
	*Hemileia vastatrix* (Coffee leaf rust)	18–26 °C and precipitation greater than 3 mm/day	Increase
	*Phoma* spp. (Phoma leaf spot)	16–20 °C and precipitation greater than 4 mm/day	Reduce
Maize—[181]	*Colletotrichum graminicola* (Anthracnose)		Increase
	*Fusarium graminearum* (Red ear rot)		Increase
	*Peronosclerospora sorghi* (Downy mildew), *Puccinia sorghi* (Common rust) *Exserohilum turcicum* (Northern corn leaf blight)	15–23 °C and RH > 60%	Increase
	*Puccinia polysora* (Polysora rust), *Physopella zeae* (Tropical rust), *Cercospora zeae-maydis* (Cercospora leaf spot, leaf streak), *Bipolaris maydis* (Leaf blight, Southern maize leaf blight)	24–32 °C and RH > 75%	Reduce
	*Stenocarpella macrospora*, *Stenocarpella maydis* (White ear rot), *Fusarium verticillioides*, *Fusarium subglutinans* (Pink ear rot), *Pythium aphanidermatum* (Stalk rot)		Remain similar
	*Ustilago maydis* (Common smut), and *Macrophomina phaseolina* (Stalk rot)	24–32 °C and water deficit	Increase
	*Erwinia chrysanthemi* (Soft rot), *E. carotovora* pv. *zeae* (Stalk rot) and *Pseudomonas alboprecipitans* (Bacterial leaf blight)	>32 °C and high humidity	Reduce
Rice—[182]	*Pyricularia grisea* (*P. oryzae) (*Rice blast)	20–30 °C	Reduce in Midwest of Brazil, and increase in Rio Grande do Sul
	*Monographella albescens* (Syn. *Metasphera albscens*) (Leaf scald)	Wetting the leaves	Increase
	*Bipolaris oryzae*, *Alternaria padwickii*, *P. grisea*, *Monographella albescens*, *Sarocladium oryzae*, *Phoma sorghina*, *Drechslera*, *Curvularia*, *Nigrospora*, *Fusarium*, *Coniothyrium*, *Epicoccum*, *Pithomyces*, *Chetomium*, *Pseudomonas*, *Erwinia* (Sheath blight, grain blight)	High temperatures, high RH and low soil fertility	Increase
	*Rhizoctonia solani* (Sheath blight)	28–32 °C and UR ± 95%	Increase
Sorghum—[183]	*Claviceps africana* (Ergot)	20–25 °C and UR > 80%	Reduce
	*Colletotrichum sublineolum* (Anthracnose)	22–30 °C and high RH	Increase
	*Exserohilum turcicum* (Northern leaf blight)	18–27 °C and wetting of the leaves	Increase
	*Fusarium moniliforme* (Fusarium head blight, root and stalk rot)	25–35 °C and high soil moisture	
	*Gloeocercospora sorghi* (Zonate leaf spot)	28–30 °C and high RH	
	*M. phaseolina* (Charcoal rot)	35–37 °C and low soil moisture	
	*P. sorghi* (Downy mildew)	21–23 °C and wetting of the leaves	Reduce
	*Puccinia purpurea* (Rust)	26–29 °C	Increase
	*Ramulispora sorghi* (Oval leaf spot)	28 °C and high RH	
Soybean—[184]	*Phakopsora pachyrhizi* (Asian soybean rust)	20–25 °C and wetting of the leaves	Reduce
Sugarcane—[185]	*Puccinia melanocephala* (Sugarcane rust)	High RH	Tendency of small influence on the disease
	*Ustilago scitaminae* (Smut)		
	*Xanthomonas albilineans* (Leaf scald)		
	*Mycovellosiella koepkei* (Yellow spot)	28 °C and RH > 80%	The disease does not find favorable conditions
	*Pothvirus—*SCMV (Streak mosaic)	Above average rains	Reduce
Winter cereals in southern Brazil—[186]	*Bipolaris sorokiniana* (Brown blotch or spot)	20–25 °C and >18 h and wetting of the leaves	Increase
	*B. sorokiniana* (Common root rot)	20–25 °C and >18 h and wetting of the leaves	Remain similar
	*Blumeria graminis* (Powdery mildew)	15–22 °C	Increase
	*Drechslera tritici-repentis* (Yellow spot)	20 °C and > 24 h wetting of the leaves	Reduce
	*Gaeumannomyces gramins* var. *tritici* (Take-all)	12–18 °C	Reduce
	*Gibberella zeae* (Fusarium head blight)	25–30 °C and >48 h wetting of the leaves	Reduce
	*Puccinia triticina* (Leaf rust)	15–20 °C and >10 h wetting of the leaves	Reduce
	*Puccinia graminis* (Stem rust)	15–30 °C and >10 h wetting of the leaves	Reduce
	*P. grisea* (Blast)	21–27 °C and 10–14 h wetting of the leaves	Increase
	*Septoria tritici* (Septoria tritici blotch)	22–26 °C and 72–96 h wetting of the leaves	Reduce
	*Septoria nodorum* (Glume blotch)	20–24 °C and 48–72 h wetting of the leaves	Reduce
**Forest**			
Black wattle—[187]	*Phytophthora nicotianae* (Gummosis)	24–28 °C	Increase
Eucaliptus in São Paulo state—[188]	*Puccinia psidii* (Rust)	Mild temperatures, high RH and long leaf wetness	Reduce
Eucalyptus—[189]	*Botrytis cinerea* (Gray mold)	20–24 °C and high RH	Remain similar
	*Ceratocystis fimbriata* (Ceratocystis wilt)	18–28 °C and high RH	Increase
	*Chrysoporthe cubensis* (Canker)	≥23 °C and precipitation ≥ 1200 mm/year	Increase
	*Coniothyrium eucalypti* (Coniothyrium canker)	Hydric stress	Remain similar
	*Cylindrocladium* spp. (Leaf spot, blight)	High temperature and RH. Wetting of the leaves	Increase
	*Erythricium salmonicolor* (Pink disease)	Precipitation ≥ 1200 mm/year	Remain similar
	*Hypoxylon* spp. (Black stromata)	30 °C and high RH	Increase
	*Oidium eucalypti* (Powdery mildew)	20–25 °C and high RH	Remain similar
	*P. psidii* (Rust)	18–25 °C and wetting of the leaves	Remain similar
	*Quambalaria eucalypti* (Leaf and shoot blight)	27 °C and high RH	Increase
	*Ralstonia solanacearum* (Bacterial wilt)	28–30 °C and high RH	Increase
	*R. solani*		Increase
	*Teratosphaeria nubilosa* (Mycosphaerella leaf)		Remain similar
	*X. axonopodis* (Bacterial leaf blight)	26–30 °C and wetting of the leaves	Increase
Pine—[190]	*Cylindrocladium pteridis* (Pine needle blight)	30–33 °C and high precipitation	Increase
	*Sphaeropsis sapinea* (Sphaeropsis blight, Tip blight)	24–26 °C and high RH	Increase
Rubber tree in São Paulo state—[191]	*Ceratocystis frimbriata* (Moldy rot)	Low temperature e and high RH	Reduce
	*Colletotrichum gloeosporioides* (Panel anthracnose)	Low temperature	Reduce
	*C. gloeosporioides* (Anthracnose)	21 °C and RH > 90%	Reduce
	*F. moniliforme* (Bark dryness)		Increase
	*Hevea pauciflora* (Pink disease)		Increase
	*Lasiodiplodia theobromae* (Stem diseases)		Increase
	*Microcyclus ulei* (Southern American leaf blight)	Prolonged wetness, RH > 95% for 10 h	Reduce
	*Oidium heveae* (Powdery mildew)		Increase
	*Phytophthora citrophthora (*Patch canker)	Mild temperature and high RH	Reduce
Fruits			
Banana—[192]	*F. oxysporum* f. sp. *cubense* (Panama disease, Fusarium wilt)		Increase
	*Mycosphaerella fijiensis* (Black sigatoka)	25–28 °C and high RH	Increase in South and Vale do Ribeira Valley in São Paulo state, and reduce in Amazon state
	*R. solanacearum* race 2 (Moko)		Reduce
Cashew—[193]	*C. gloeosporioides (*Anthracnose)	Rain and high RH	Increase
	*Lasiodiplodia theobromae* (Gummosis)	Hydric stress	
	*Oidium anacardii* (Powdery mildew)	26–28 °C	
	*Pilgeriella anacardii* (Black mould)	Rain	
	*Xanthomonas campestris* pv. *mangifereaeindicae* (Bacterial leaf, Fruit spot)		
Citrus in São Paulo state—[194]	*Colletotrichum acutatum* (Citrus postbloom frui drop disease)	23–27 °C and leaf wetness between 10 to 12 h	Remain similar
	*Guignardia citricarpa* (*Phyllosticta citricarpa* (Citrus black spot)	21–32 °C and leaf wetness between 24 to 48 h	Increase
	*Candidatus* Liberibacter spp. (Huanglongbing = Greening)	High temperatures favor the *Diaphorina citri* vector	In the North and Northwest regions, the tendency is to remain similar its importance. In the central and southern regions, the tendency is for an increase in importance
	*Xanthomonas axonopodis* pv. *citri* (Citrus canker)	30–35 °C and wetting of the leaves for 24 h	Increase
	*Xylella fastidiosa* (Citrus variegated chlorosis)	High temperatures and water deficit.	Increase
Coconut—[195]	*Bipolaris incurvata* (Leaf spot, Bipolaris leaf blight)	18–27 °C and high RH	Reduce
	*Botryosphaeria cocogena* (Leaf blight)	Rain between 25–80 mm	
	*Camarotella torrendiella* and *Camarotella acrocomiae* (Tar spot, black leaf spot)	High RH	
	*Phytophthora* spp. (Bud rot, nutfall)	25–28 °C and high RH in poorly drained soils	
	*Thielaviopsis* (*Ceratocystis) paradoxa* (Stem bleeding disease)		Increase
Grape—[196]	*Elsinoe ampelina* (Anthracnose)	24–26 °C and RH > 90%	Remain similar
	*Phakopsora euvitis* (Rust)	16–30 °C and wetting of the leaves	
	*Plasmopara viticola* (Downy mildew)	20–25 °C, high RH and wetting of the leaves	
	*Phomopsis viticola* (Leaf spot, Phomopsis cane)	23–25 °C and wetting of the leaves	
	*Pseudocercospora vitis* (Leaf blight)	High temperature and RH	
	*Uncinula necator* (Powdery mildew)	25 °C and RH between 40-60%	Remain similar, with an increasing trend in some regions
Mango—[197]	*Ceratocystis fimbriata* (Mango wilt)	High temperature and rainy periods	Remain similar
	*C. gloeosporioides* (Anhtracnose)	>25 °C, RH > 95% and wetting of the leaves	Increase
	*Elsinoe (Sphaceloma) mangiferae* (Mango scab)	High RH	Remain similar
	*Fusarium* spp. (Mango malformation)	Rain	
	*L. theobromae* (Stem end rot, Die back, Gummosis)	27–32 °C and RH > 80%	Increase
	*Oidium mangiferae (Erysiphe polygoni)* (Powdery mildew)	20–25 °C and RH between 20–65%	Increase in São Paulo, Minas Gerais, Espírito Santo and Bahia states
	*X. campestris* pv. *mangiferaindica* (Bacterial black spot)	High temperature and rainy periods	Remain similar
Melon—[198]	*C. gloeosporioides* (Anthracnose)	21–27 °C and high RH	Increase
	*Corynespora cassiicola* (Corynespora leaf disease)	25–35 °C and high RH	Increase
	*Didymella bryoniae* (Gummy stem blight)	22–32 °C and high RH	Increase
	*Monosporascus cannobalus* and *M. phaseolina* (root rot, vine decline, sudden wilt, sudden death, melon collpase)	30–35 °C, low soil moisture and and alkaline pH	Disease has assumed significant importance at the moment. Tendency of indefinite importance.
	*Phodosphaera xanthii*, *Golovinomyces cichoracearum* (Powdery mildew)	10–32 °C and high RH	Remain similar
	*Pseudoperonospora cubensis* (Downy mildew)	5–30 °C and water film by > 6 h	Increase
	*Acidovorax avenae* subsp. *citrulli* (Bacterial fruit blotch)	±26 °C and high RH	Increase
Papaya—[199]	*Asperisporium caricae* (Black spot)	23–27 °C	Increase in Espírito Santo state, and will reduce in other regions
	*C. gloeosporioides* (Anthracnose, charcoal spot)	21–27 °C, RH > 97 and wetting of the leaves	Increase
	*Corynespora cassiicola* (Corynespora target spot)	High RH and temperature	
	*L. theobromae* (Stem end rot)		Remain similar
	*Oidium caricae*, *Ovulariopsis papayae* (Powdery mildew)	15–20 °C and RH between 60–70%	Reduce
	*Phytophthora palmivora* and *Phytophthora parasitica* (Papaya fruit rot)	25 °C and high soil moisture	Increase in irrigate crops
	*Phoma caricae papayae* (Leaf spot)	Rainy days	Reduce
	*Pythium*, *R. solani*, *Fusarium* sp. and *Phytophthora* sp. (Damping-off)	High temperature and RH	
	*Papaya lethal yellowing virus* (PLYV)		Remain similar
Pineapple—[200]	*Fusarium subglutinans* f. sp. *ananas* (Gommusis)	15–22 °C and high precipitation	Increase
	*P. nicotianae* var. *parasitica* (Heart rot)	25–36 °C and high precipitation	
	*P. cinnamomi* (Root rot)	19–25 °C	Reduce
Stone fruit—[201]	*Armillaria mellea* (Armillaria root rot)		Increase
	*Botryosphaeria dothidea* (Gommusis)		
	*Cladosporium carpophilum* (Scab)	25–30 °C and high RH	Increase in South, and remain similar in Southeast region
	*Glomerella cingulata* (Anthracnose)	25–30 °C and high RH	Increase in South, and remain similar in Southeast region
	*Monilinia fructicola* (Brown rot)	25 °C and high RH	Increase
	*Phomopsis amygdali* (Twig canker)	27–29 °C	
	*Phytophthora* spp. (Crown rot)	30–32 °C	
	*Rhyzopus stolonifer* (Rhyzopuys rot)	15–23 °C and high RH	
	*Taphrina deformans* (Peach leaf curl)	18–20 °C and RH > 95%	
	*Tranzschelia discolor* (Rust)	18–26 °C	
	*Wilsonomyces carpophylus* (Shot hole)	15–20 °C	
	*Xanthomonas arboricola* pv. *pruni* (Bacterial spot	30 °C and wetting of the leaves	
	*Xyllela fastidiosa* (Phony peach disease)	20–25 °C	
Strawberry—[202]	*B. cinerea* (Gray mold)	20 °C, high RH and wetting of the leaves	Remain similar
	*Colletotrichum acutatum* (Anthracsone fruit rot)	18–23 °C	Reduce
	*Colletotrichum fragariae* (Anthracnose)	High temperature and RH	Increase during rainfall
	*Mycosphaerella fragariae*, *Diplocarpon earlianum*, *Dendrophoma obscurans*, *Pestalotiopsis longisetula* (Leaf spot)	25–30 °C and high RH	Increase
	*Phytophthora cactorum*, *S. sclerotiorum* and *R. solani* (Root rot, fruit rot)	15–22 °C, high RH and rain	Reduce
	*Podosphaera aphanis* (*Sphaerotheca macularis*) (Powdery mildew)	15–30 °C	Increase
	*R. solani*, *Fusarium*, *Pythium ultimum*, *Phytophthora* (Root rot)	25–27 °C and high soil moisture	Increase in soils with excessive moisture
	*Verticillium dahliae* (Verticillium wilt)	20–25 °C and hydric stress	Increase
	*Xanthomonas fragariae* (Bacterial angular leaf spot)	18–22 °C and high RH	
	Redness	Factors that cause plant stress	
Vegetables			
Brassicas—[203]	*Alternaria brassicae* and *Alternaria brassicicola* (Alternaria leaf spot)	20–28 °C and high RH	Remain similar
	*Peronospora parasitica* (Downy mildew)	14–18 °C and high RH	Reduce
	*Plasmodiophora brassicae* (Clubroot)	20–25 °C and high soil moisture	Increase
	*Pseudomonas syringae* pv. *maculicola* (Bacterial leaf spot)	22–25 °C and high RH	Remain similar in South and Southeast, and will reduce in other regions
	*R. solani* (Wirestem)	25–30 °C and high soil moisture	Increase
	*Sclerotinia sclerotiorum* (White mould)	15–20 °C and high RH	Reduce
	*Sclerotium rolfsii* (Stem rot)	22–30 °C	Increase
	*Pectobacterium carotovorum* subsp. *carotovorum* (Soft rot)	High soil moisture and high temperature	Increase
	*X. campestris* pv. *campestres* (Black rot)	28–30 °C and high RH	Remain similar of favorability but with an upward trend
Lettuce—[204]	*Pythium* spp. *(*Damping-off)	20–30 °C and high RH	Increase in hydroponic systems
	*Bremia lactucae* (Downy mildew)	18–20 °C, high RH and wetting of the leaves	Increase in Rio Grande do Sul and Santa Catarina states during the winter, and will reduce with increase in temperature
	*Cercospora longissima* (Cercospora leaf spot)	20–30 °C, high RH and wetting of the leaves	Increase
	*Erysiphe cichoracearum* (Powdery mildew)	22–30 °C	Increase
	*F. oxysporum* f. sp. *lactucae* (Fusarium wilt)	>27 °C	Increase between October and May
	*R. solani* (Damping-off)	25–30 °C and high RH	Increase between December and May
	*S. sclerotiorum*, *S. minor* (Leaf drop)	15–21 °C and wetting of the leaves > 12h	Reduce
	*S. rolfsii* (Southern blight)	25–35 °C and high RH	Increase between December and May
	*Septoria lactucae* (Septoria leaf spot)	10–25 °C	Remain similar of the current winter scenario for Rio Grande do Sul, Santa Catarina, Paraná, Rio de Janeiro, and Minas Gerais states with the use of irrigation. Reduction for other periods and regions.
	*Thielaviopsis basicola* (Black root rot)	23–30 °C	Increase
	*P. carotovorum* (Bacterial soft rot)	25–30 °C and high RH	Increase between October and March
	*Pseudomonas cichorii*, *X. axonopodis* pv. *vitians* (Bacterial leaf spot)	18–25 °C, high RH and wetting of the leaves	Reduce
Onion—[205]	*Alternaria porri* (Purple blotch)	21–30 °C and wetting of the leaves	Increase
	*Botrytis squamosa* (Botrytis leaf blight)	12–16 °C and high RH	Reduce
	*Colletotrichum circinans* (Anthracnose)	26 °C	Increase in times with high temperatures
	*C. gloeosporioides* f. sp. *cepae* (Mal-de-sete-voltas)	23–30 °C and high RH	Increase
	*Fusarium oxysporum* f. sp. *cepae* (Fusarium basal plate rot)	20–30 °C and high soil moisture	Increase in times with high rainfall
	*Peronospora destructor* (Downy mildew)	12 °C and RH > 80%	Reduce
	*Pyrenochaeta terrestres* (Pink root)	24–28 °C and high soil moisture	Increase during rainfall
	*P. nicotinae* (Phytophthora neck)	High soil moisture and > 25 °C	Increase
	*Sclerotium cepivorum* (White rot)	Soil temperature between 10–20 °C	Reduce
	*Burkholderia cepacia* (Sour skin)	30–35 °C and high RH	Increase
	*P. carotovorum* subsp. *carotovorum* (Soft rot)	20–30 °C and high RH	Increase
Potato—[206]	*Alternaria solani* (Early blight)	20–24 °C	Remain similar, with a tendency to increase.
	*Helminthosporium solani* (Silver scab)	High soil moisture (>90%)	Increase
	*Phytophthora infestans* (Late blight)	Zoospore production: 8–18 °C; sporangia germination: 18–25 °C. High humidity	Remain similar, with a tendency to reduce
	*R. solani* (Rhizoctonia)	<20 °C	Remain similar, with a tendency to reduce
	*Spongospora subterrânea* (powdery scab)	Soil temperature between 11–18 °C, with high humidity	Reduce
	*S. sclerotiorum* (White mold)	15–21 °C and high humidity	Reduce
	*S. rolfsii* (Crown rot, Southern blight)	28–30 °C and high soil moisture	Increase
	*Pectobacterium* (*Erwinia*) (Cinnamon black, and soft rot)	>30 °C	Increase
	*R. solanacearum* (Bacterial wilt)	Around 30 °C and high soil moisture	Increase
	*Streptomyces* (Commom scab)	25–30 °C and low soil moisture	Remain similar, with a tendency to reduce
	*Meloidogyne incognita* (root knot nematode)	25–32 °C	Remain similar, with a tendency to increase.
Pepper—[207]	*B. cinerea* (Gray mold)	18–23 °C and RH between 90%–95%	Reduce
	*Cercospora capsici* and *Stemphylium solani* (Leaf spot)	23–27 °C and RH > 90%	Remain similar, with a tendency to reduce
	*Colletotrichum* (Anthracnose)	20–30 °C and high RH	Remain similar, with a tendency to reduce
	*Oidiopsis taurica* (Powdery mildew)	10–35 °C and RH between 85%–95%	Remain similar, with a tendency to increase
	*Phytophthora capsici* (Phytophthora blight)	22–28 °C and high RH	Remain similar, with a tendency to reduce
	*S. sclerotiorum* (White mold)	16–22 °C and high RH	Reduce
	*S. rolfsii* (Southern blight)	25–30 °C and high RH	Remain similar, with a tendency to increase
	*C. michiganesis* subsp. *michiganensis* (Bacterial canker)	24–28 °C and high RH	Reduce
	*Erwinia carotovora* subsp. *carotovora* (Soft rot)	28–30 °C and high RH	Increase
	*R. solanacearum* (Bacterial wilt)	30–35 °C and high soil moisture	Increase
	*X. campestris* pv. *vesicatoria* (Bacterial spot)	22–28 °C and RH between 95%–100%	Remain similar, with a tendency to increase
	*Tomato mosaic virus* (ToMV), *Tobacco mosaic virus* (TMV), *Pepper mild mottle virus* (PMMoV) (Mosaics—viruses transmitted mechanically)		Remain similar
Tomato—[208]	*A. solani* (Early blight)	25–32 °C and free water on the surface of the leaves	Remain similar, with a tendency to increase
	*B. cinerea* (Gray mold)	18–23 °C, RH > 90%	Reduce
	*F. oxysporum* f. sp. *lycopersici* (Fusarium wilt)	21–33 °C	Increase
	*Leveilula taurica* (Powdery mildew)	High temperature and low RH	Increase
	*P. infestans* (Late blight)	12–18 °C and rain > 24 h	Reduce
	*Septoria lycopersici* (Septoria leaf spot)	20–25 °C, mild temperatures and abundant rainfall	Remain similar, with a tendency to reduce
	*Stemphylium solani* (Gray leaf spot)	25–28 °C and UR > 80%	Remain similar, with a tendency to increase
	*S. sclerotiorum* (White mold)	15–21 °C and high humidity	Reduce
	*S. rolfsii* (Southern blight)	25–35 °C and high humidity	Increase
	*Verticillium albo-atrum*, *V. dahliae* (Verticillium wilt)	22–25 °C	Reduce
	*Clavibacter michiganensis* subsp. *michiganensis* (Bacterial canker)	18–25 °C and high RH	Reduce
	*Erwinia* spp. (Soft rot)	25–30 °C and RH around 100%	Reduce
	*Pseudomonas corrugata* (Pith necrosis)	Mild night temperatures and high RH	Reduce
	*P. syringae* pv. tomato (Bacterial speck)	18–25 °C and high RH	Reduce
	*R. solanacearum* (Bacterial wilt)	24–35 °C and high soil moisture	Increase
	*Xanthomonas* spp. (Bacterial spot)	24–30 °C and high RH	Increase
	*Tomato mosaic virus*		Remain similar

Obs.: Projections of future climate conditions based on the 3rd IPCC Report in Ghini and Hamada [178] *; considering 2020s, 2050s, 2080s; and based on the 4th IPCC Report in Ghini et al. [209], considering 2050s and 2080s. (*) The English version of this reference was published in Ghini and Hamada [210] and cited considering it.

**Table 2:** Effects of climate change on different pathosystems based on risk analysis using maps of geographic and temporal distribution of Brazil.

Host	Pathogen (Disease)	Effects of Climate Change on Future	References
Banana	*Mycosphaerella fijiensis* (black Sigatoka)	There will be a reduction in the favorable area	[120,211]
Cacao	*Moniliophthora roreri* (frosty pod rot of cocoa)	Favorability will be increased	[212]
	*M. roreri* (Moniliasis)	The potential risk will be reduced	[213]
Coffee	*Hemileia vastatrix* (coffee leaf rust)	The severity will increase with the reduction in the incubation period in the states of Minas Gerais and São Paulo	[214]
	*H. vastatrix* (coffee leaf rust)	The incubation period will be reduced	[121]
	*Meloidogyne incognita* (root disease)	The infestation of the nematode will be increased	[215]
	*Mycena citricolor* (American leaf spot)	There will be a reduction in favorability for the disease in future decades, except in southern Brazil during May and July	[216]
	*Phoma* sp. (Phoma leaf spot)	There will be a reduction in some areas, but there will still be potential risk in the Southern region	[217]
Common beans	*Fusarium solani* species complex (root rot)	Strong convergence on the environmental requirements of both the host and the disease development. Climate change will probably move the disease toward cooler regions	[218]
Eucalyptus	*Puccinia psidii* (rust)	There will be a reduction in the favorable area	[219]
Grape	*Glomerella cingulata* (ripe rot) and *Botrytis cinerea* (gray mold)	There will be a reduction in the favorable area in Brazilian Northeast	[220]
	*Plasmopara viticola* (downy mildew)	Favorability will be increased in Rio Grande do Sul and Santa Catarina states. There will be a reduction in the favorability in São Francisco Valley. For Northern Paraná state and Eastern São Paulo state, the condition will be the same as the current ones	[221]
	*Uncinula necator* (powdery mildew)	There will be an increase in the favorable area	[222]
Papaya	*Asperisporium caricae* (smallpox)	There will be a reduction in the favorable area	[223]
Peanut	*Cercosporidium personatum* (black spot)	There will be an increase in the favorable area	[224]

**Table 3:** Effects of increased CO_2_ under controlled conditions on the incidence and severity of different pathosystems in Brazil.

Host	Pathogen (Disease)	Effects of Increased CO_2_	References
Coffee	*Hemileia vastatrix* (coffee leaf rust)	The severity was reduced	[231]
	*H. vastatrix* (coffee leaf rust) *Cercospora coffeicola* (Cercospora leaf spot)	There was no significant effect of CO_2_ on diseases incidence	[76]
	*Leucoptera coffeella* (leaf miner)	The incidence of leaf minor was lower under elevated CO_2_	[76]
	*H. vastatrix* (coffee leaf rust)	The incidence of coffee leaf was the same in elevated and ambient CO_2_	[126]
	*L. coffeella* (leaf miner)	The incidence of leaf minor was lower under elevated CO_2_	[126]
Eucalyptus	*Cylindrocladium candelabrum* (leafspot)	The severity and incidence were reduced	[226]
	*Puccinia psidii* (rust)	The severity was reduced, and growth plant was stimulated	[73]
	*Ceratocystis fimbriata*	The severity was reduced, and growth plant was stimulated	[225]
Melon	*Oidium* sp. (powdery mildew)	The severity will be reduced, and the incubation period will be increased	[227]
Rice	*Bipolaris oryzae* (brown spot)	The severity was reduced	[232,233]
	*Magnaporthe oryzae* (rice blast)	The disease was more severe	[230]
Soybean	*Microsphaera diffusa* (powdery mildew)	The severity was increased	[229]
	*Phytophthora sojae* (stem canker)	Plant defense responses was changed	[228]
